# The Minnesota living with heart failure questionnaire: comparison of different factor structures

**DOI:** 10.1186/s12955-016-0425-7

**Published:** 2016-02-17

**Authors:** Amaia Bilbao, Antonio Escobar, Lidia García-Perez, Gemma Navarro, Raul Quirós

**Affiliations:** Research Unit, Basurto University Hospital (Osakidetza), Bilbao, Bizkaia Spain; Health Service Research Network on Chronic Diseases (REDISSEC), Bilbao, Bizkaia Spain; Evaluation Service, Dirección del Servicio Canario de la Salud, Tenerife, Canary Islands Spain; Epidemiologic Unit, Corporació Parc Tauli Clinic, Sabadell, Barcelona, Spain; Department of Internal Medicine, Costa del Sol Hospital, Marbella, Málaga, Spain

**Keywords:** Minnesota living with heart failure questionnaire, Heart failure, Health-related quality of life, Factor analysis, Rasch analysis, Psychometric properties

## Abstract

**Background:**

The Minnesota Living with Heart Failure Questionnaire (MLHFQ) is one of the most widely used health-related quality of life questionnaires for patients with heart failure (HF). It provides scores for two dimensions, physical and emotional, and a total score. However, there are some concerns about its factor structure and alternatives have been proposed, some including a third factor representing a social dimension. The objectives of the present study were to analyze the internal structure of the MLHFQ and the unidimensionality of the total score, and to compare the different factor structures proposed.

**Methods:**

The MLHFQ was given to 2565 patients with HF. The structural validity of the questionnaire was assessed by confirmatory factor analysis (CFA), and Rasch analysis. These two approaches were also applied to the alternative structures proposed.

**Results:**

The CFA results for the hypothesized model of two latent factors and the Rasch analysis confirmed the adequacy of the physical and emotional scales. Rasch analysis for the total score showed only two problematic items. The results of the CFA for other two-factor structures proposed were not better than the results for the original structure. The Rasch analyses applied to the different social factors yielded the best results for Munyombwe’s social dimension, composed of six items.

**Conclusions:**

Our results support the validity of using the MLHFQ physical, emotional and total scores in patients with HF, for clinical practice and research. In addition, they confirmed the existence of a third factor, and we recommend the use of Munyombwe’s social factor.

## Background

Heart failure (HF) is one of the most important health problems in terms of prevalence, morbidity, mortality and health service use [1]. It affects around 2 to 3 % of the population and the prevalence increases with age, affecting as much as around 10 to 20 % of the population over 65 years old [1–3]. In developed countries, the prevalence of HF is increasing due to population aging, longer survival of patients and effectiveness of secondary prevention [4, 5]. Projections indicate that the prevalence of HF will increase as much as 46 % from 2012 to 2030 [1]. In brief, HF is a common disease with a huge impact on the prognosis and lifestyle of patients and a growing challenge for health policy makers [6].

The health-related quality of life (HRQoL) of patients with HF is an important outcome as it reflects the impact of HF on their daily lives [7, 8]. Instruments to assess HRQoL provide a way to explore the perceptions of patients about how HF affects their daily lives and wellbeing, providing information that cannot be obtained directly from clinical measurements [5]. In recognition of this, improving the HRQoL has emerged as an important treatment goal [4, 9, 10].

Various specific HRQoL questionnaires for patients with HF have become regarded as important assessment tools in recent decades [2, 5, 11, 12]. Among these, one of the most widely known and used is the Minnesota Living with Heart Failure Questionnaire (MLHFQ) [10, 11, 13], which has been translated and culturally adapted into at least 34 languages, and has demonstrated good psychometric properties in numerous studies [2, 5, 7, 11, 14–20]. However, there are some concerns about its factor structure and the homogeneity of items [7, 10, 13, 17]. Several authors have even proposed different factor structures [4, 7, 15, 18, 21]. When reviewing validation studies, we encountered certain problems and weaknesses. Firstly, while some authors obtained similar factor structures to the original developers of the questionnaire [18, 22, 23], others obtained two-factor structures but disagreed on certain items [7], and various even extracted structures of three factors, with a new social subscale [4, 15, 16, 18, 21], but disagree on the items that make up the third factor. Secondly, in clinical practice, the MLHFQ is commonly used to generate a total score, which assumes that the total scale is unidimensional, but we found only two studies analyzing a single-factor structure [15, 18], and they differ in their conclusions. Lastly, most studies that analyze the structural validity of the instrument have been carried out from the perspective of classical test theory (CTT) [4, 7, 16–19, 21, 22], and more specifically, using techniques of exploratory factor analysis (EFA) rather than confirmatory factor analysis (CFA). Considering all this, we posed the following questions. Is the total score unidimensional? Does the questionnaire have a two-factor structure? Or is there a third factor representing a social dimension? And if so, which of the social factors proposed is the most appropriate?

Therefore, the objectives of the present study were: 1) to conduct a validation study of the MLHFQ, analyzing the internal structure using both CTT and item response theory (IRT); 2) to compare different factor structures proposed by other authors; and 3) to assess other psychometric properties including known-groups validity, convergent validity, and reliability of the different social factors proposed.

## Methods

### Study population

The current study included patients recruited from 13 participating hospitals of the Spanish National Health Service between December 2008 and May 2013. Consecutive patients hospitalized for HF in cardiology or internal medicine departments of the participating hospitals during the study period were invited to participate. Patients were excluded if they had any organic or psychiatric disorder that might hinder completion of questionnaires. The study was approved by the corresponding institutional review boards.

### Measurements

All eligible patients were given a letter informing them about the study and asking for their voluntary participation. In addition, they were given the MLHFQ [13], and the 12-item Short Form Health Survey (SF-12) [24, 25] for completion during hospitalization. Six months after hospitalization, the same questionnaires were sent by mail to patients at home for completion and return by mail. A reminder letter was sent to patients who had not replied within 15 days. Sociodemographic and clinical data were also collected.

The MLHFQ is a self-administered disease-specific questionnaire for patients with HF [13], comprising 21 items rated on six-point Likert scales, representing different degrees of impact of HF on HRQoL, from 0 (none) to 5 (very much). It provides a total score (range 0–105, from best to worst HRQoL), as well as scores for two dimensions, physical (8 items, range 0–40) and emotional (5 items, range 0–25). The other eight items (of the total of 21) are only considered for the calculation of the total score. The MLHFQ has been translated into and validated in Spanish [5, 19].

The SF-12 is a generic questionnaire for assessing HRQoL [24, 25] comprising 12 items and two summary scales: the physical and mental component summary (PCS and MCS). The scores for these components range from 0 to 100, with higher scores indicating better health status. The SF-12 has been translated into and validated in Spanish [26].

### Statistical analysis

The unit of analysis was the patient. If patients had more than one hospitalization during the study period, only the first was considered. The descriptive statistical analysis was based on frequency tables, and means and standard deviations (SDs).

#### Construct validity

To study the structural validity of the questionnaire, two different approaches were used. First, CFA for categorical data was used to confirm the hypothesis that 13 items on the questionnaire reflected two dimensions, physical and emotional, as proposed by the original developers [13]. Second, Rasch analysis within IRT models was used to assess each specific dimension of the questionnaire, as well as the total scale, for unidimensionality. These two approaches were also applied to different factor structures proposed by other authors [4, 7, 15, 16, 18, 21], to compare the structures (Appendix 1).

Regarding the CFA, the robust unweighted least squares estimator was used, and three fit indices were calculated [27–31]: the root mean square error of approximation (RMSEA), for which a value <0.08 was considered acceptable; and the Tucker-Lewis Index (TLI) and Comparative Fit Index (CFI), for both of which >0.90 was considered acceptable. We also examined factor loadings, and those ≥0.40 were considered acceptable. For the comparison of different factor structures, as the models are non-nested, we used the Akaike Information Criterion (AIC), with lower values indicating a better fit.

In relation to the IRT models, we used the polytomous Rasch rating scale model because the response scales of the questionnaire are ordinal with six response options [32–35]. We applied the Rasch method to the total score and each specific dimension separately to check whether the scales were unidimensional [36] as this is a fundamental requirement for construct validity [37]. Unidimensionality was assessed with two fit indices, namely the mean square information-weighted statistic (infit) and the outlier-sensitive statistic (outfit), with values between 0.7 and 1.3 indicating a good fit [38], and a principal component analysis (PCA) of the residuals. Unidimensionality was considered violated if, besides the first factor, other factors had eigenvalues >3 [39]. We evaluated the ability of the MLHFQ to define a distinct hierarchy of items along each measured dimension by means of an item separation index [36]. A value of >2.0 is comparable to a reliability of 0.80 and considered acceptable. To detect the presence of differential item functioning (DIF), which occurs when different groups within the sample respond in a different manner to an individual item [32], we compared different levels of the trait by sex and age group (≤65 vs. >65 years). A Welch’s *t* statistically significant at *P* < 0.05, and a difference in difficulty of ≥0.5 logit were considered to be noticeable DIF [39]. Residuals correlations between items within a scale were examined for local dependency. Correlations >0.5 between item residuals may indicate that responses to one item may be determined by those to another [40]. The functioning of rating scale categories was also examined for each item. A clearly progressive level of difficulty across the item categories was considered adequate [39].

#### Convergent validity

We assessed convergent validity of the different social factors by analyzing the relationship between the MLHFQ social scale and SF-12 scores with Spearman’s correlation coefficient.

#### Known-groups validity

Known-groups validity of the different social factors was examined by comparing the MLHFQ social subscale scores 6 months after discharge among groups based on whether or not the patient had (a) attended the emergency department or (b) had any readmissions during the previous 6 months. For this analysis, we used data from the 6-month follow-up, and we used t-tests or non-parametric Wilcoxon tests. We hypothesized that patients who had attended the emergency department or had any readmissions would obtain worse MLHFQ social scores at 6 months. Furthermore, to assess the magnitude of group differences, the effect size was calculated as the mean difference divided by the pooled standard deviation. Cohen’s benchmarks were used to classify the magnitude of effect sizes: <0.20 being considered not significant; 0.20 to 0.49 small, 0.50 to 0.79 moderate, and ≥0.80 large [41].

#### Reliability

We assessed internal consistency using Cronbach’s alpha coefficient [42]. A coefficient >0.70 was considered acceptable [43].

All effects were considered statistically significant at *P* < 0.05. The statistical analyses were performed with SAS for Windows (version 9.2; SAS Institute, Cary, NC), Mplus (version 6.1; Muthén et al., 1998–2010), and Winsteps (version 3.69.1.4; John M. Linacre, Chicago).

## Results

During the recruitment period, 2565 patients hospitalized for HF fulfilled the selection criteria, agreed to participate and completed the baseline questionnaires. Of these, 1211 (47.21 %) completed the questionnaires 6 months after discharge. Table 1 shows descriptive statistics for the sociodemographic, clinical and HRQoL data at baseline.

**Table 1:** Sociodemographic and clinical variables, and baseline MLHFQ and SF-12 scores: descriptive statistics of the sample (*N* = 2565)

Parameter	n (%)
Age, mean (SD)	77.25 (10.21)
Sex, men	1291 (50.33)
Body mass index (kg/m^2^)	
BMI < 25	234 (20.47)
25 ≤ BMI < 30	426 (37.27)
30 ≤ BMI < 35	299 (26.16)
BMI ≥35	184 (16.10)
Smoking history	
No	1232 (58.17)
Ex	710 (33.52)
Yes	176 (8.31)
Left ventricular ejection fraction	
≤45 %	888 (39.15)
>45 %	1380 (60.85)
NYHA classification at discharge	
I	89 (3.62)
II	1306 (53.07)
III	1021 (41.49)
IV	45 (1.83)
Charlson comorbidity index	
0	173 (6.88)
1	500 (19.90)
2	578 (23)
3	484 (19.26)
>3	778 (30.96)
MLHFQ score, mean (SD)	
Physical subscale	27.40 (9.05)
Emotional subscale	12.40 (7.28)
Total scale	57.85 (22.69)
SF-12 score, mean (SD)	
Physical Component Summary	29.30 (8.91)
Mental Component Summary	41.55 (11.36)

Data are expressed as frequency (percentage) unless otherwise stated. Percentages exclude patients with missing data

*SD* Standard deviation, *BMI* Body mass index, *NYHA* New York Heart Association

The scores for the MLHFQ physical subscale range from 0 to 40, the emotional subscale from 0 to 25, and the total scale from 0 to 105, with higher scores indicating worse health status. The scores for the SF-12 domains range from 0 to 100, with higher scores indicating better health status

### Construct validity

**Fig. 1:**
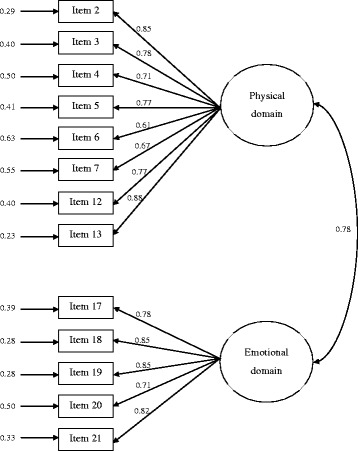
Confirmatory factor analysis of the MLHFQ physical and emotional subscales (*N* = 2565). Standardized parameters are shown

The results of the CFA for the hypothesized model of two latent factors, physical and emotional, provided satisfactory fit indices (Table 2). The RMSEA value was around 0.08, and CFI and TLI values both exceeded 0.90. All factor loadings were statistically significant (*P* < 0.001) and >0.40 (Fig. 1).

**Table 2:** Results of fit indices of confirmatory factor analysis of the different MLHFQ structures (*N* = 2565)

Models (first author)	No. of factors	χ^2^ (df)	RMSEA (90 % CI)	CFI	TLI	Factor loading (Range)	AIC
2-factor models							
Rector	2	1002.34 (60)	0.083 (0.079 − 0.088)	0.970	0.961	0.61 − 0.88	1064.34
Heo	2	1100.03 (85)	0.080 (0.076 – 0.084)	0.966	0.958	0.50 – 0.87	1170.03
3-factor models							
Ho	3	3011.42 (145)	0.103 (0.100 − 0.106)	0.909	0.892	0.35 − 0.86	3101.42
Moon	3	3711.48 (182)	0.102 (0.099 − 0.105)	0.903	0.888	0.34 − 0.86	3809.48
Lambrinou	3	2351.97 (144)	0.091 (0.087 − 0.094)	0.929	0.916	0.32 − 0.89	2483.97
Garin	3	1414.26 (112)	0.079 (0.075 − 0.083)	0.952	0.942	0.43 − 0.89	1496.26
Munyombwe	3	2687.35 (163)	0.091 (0.088 − 0.094)	0.927	0.914	0.35 − 0.87	2781.35

*χ* ^*2*^ Chi-square, *df* degrees of freedom, *RMSEA* root mean square error of approximation, *CI* Confidence interval, *CFI* comparative fit index, *TLI* Tucker-Lewis fit index, *AIC* Akaike information criterion

Regarding the results of the Rasch analysis for the physical and emotional dimensions, and the total score (Table 3), the unidimensionality was supported with infit and outfit statistics of 0.7 to 1.3, except in three items of the physical dimension: item 3 with an outfit value slightly below 0.7, item 7 with infit and outfit values slightly above 1.30, and item 6 with infit and outfit values above 1.30 (infit = 1.54 and outfit = 1.69); and the following items of the total score: items 8 and 10 with fit statistics substantially above 1.3; item 1 with an infit value slightly above 1.3 but an outfit value of 1.82; item 2 with an infit value slightly below 0.7; and item 13 with both fit statistics slightly below 0.7. However, the PCA of the residuals did not yield additional factors with eigenvalues >3, implying that the unidimensionality assumption was met. The item separation indices were >2, indicating reliability of >0.80. The presence of DIF was not detected by sex or age group, except in item 10 for the total score, this item being more difficult for men than women and for patients ≤65 years than those >65 years. Correlation coefficients between residuals were all <0.50, supporting the assumption of local independence, and the functioning of the rating scale categories was adequate.

**Table 3:** Severity levels, standard errors, and goodness-of-fit indices of the MLHFQ specific dimensions and the total score using Rasch analysis (*N* = 2565)

Items	Item description	*δ* (logit)	SE	Infit MNSQ	Outfit MNSQ
Physical					
Item 7	Relating to or doing things with friends or family difficult	1.20	0.02	1.34	1.37
Item 6	Sleeping difficult	0.50	0.02	1.54	1.69
Item 2	Resting during day	0.12	0.02	0.79	0.88
Item 4	Working around house difficult	−0.15	0.02	1.12	1.08
Item 12	Shortness of breath	−0.27	0.02	1.08	1.12
Item 5	Being away from home difficult	−0.41	0.02	0.86	0.80
Item 13	Fatigue	−0.46	0.02	0.81	0.77
Item 3	Walking or climbing stairs difficult	−0.53	0.02	0.72	0.67
Emotional					
Item 17	Feeling burden to family or friends	0.48	0.02	1.05	0.99
Item 18	Feeling a loss of self-control	0.24	0.02	0.82	0.79
Item 20	Difficulty concentrating or remembering	0.22	0.02	1.24	1.25
Item 21	Being depressed	−0.22	0.02	0.98	0.96
Item 19	Being worried	−0.73	0.02	0.95	0.88
Total					
Item 16	Side effects from medications	0.80	0.02	1.13	1.17
Item 10	Sexual activities difficult	0.65	0.02	1.84	1.91
Item 15	Medical costs	0.57	0.02	1.02	1.03
Item 8	Working to earn a living difficult	0.44	0.02	1.86	1.97
Item 17	Feeling burden to family or friends	0.42	0.01	0.94	0.91
Item 7	Relating to or doing things with friends or family difficult	0.35	0.01	0.75	0.72
Item 18	Feeling a loss of self-control	0.29	0.01	0.75	0.72
Item 20	Difficulty concentrating or remembering	0.28	0.01	1.02	1.17
Item 11	Eat less food you like	0.19	0.01	1.03	1.15
Item 9	Recreational activities difficult	0.05	0.01	1.07	1.05
Item 21	Being depressed	0.03	0.01	0.82	0.84
Item 1	Swelling in your ankles, legs	−0.07	0.01	1.37	1.82
Item 6	Sleeping difficult	−0.08	0.01	1.06	1.21
Item 14	Hospitalization	−0.20	0.02	1.11	1.17
Item 19	Being worried	−0.26	0.02	0.83	0.81
Item 2	Resting during day	−0.32	0.02	0.68	0.76
Item 4	Working around house difficult	−0.49	0.02	1.03	1.04
Item 12	Shortness of breath	−0.57	0.02	0.83	0.86
Item 5	Being away from home difficult	−0.66	0.02	0.87	0.88
Item 13	Fatigue	−0.69	0.02	0.66	0.61
Item 3	Walking or climbing stairs difficult	−0.74	0.02	0.75	0.72
Social proposed by Lambrinou					
Item 10	Sexual activities difficult	0.40	0.02	1.21	1.03
Item 8	Working to earn a living difficult	0.09	0.02	0.97	0.87
Item 9	Recreational activities difficult	−0.49	0.02	0.80	0.85
Social proposed by Garin					
Item 10	Sexual activities difficult	0.24	0.02	1.03	0.90
Item 15	Medical costs	0.16	0.02	1.33	1.43
Item 8	Working to earn a living difficult	0.01	0.02	0.97	0.87
Item 9	Recreational activities difficult	−0.41	0.02	0.68	0.67
Social proposed by Munyombwe					
Item 16	Side effects from medications	0.40	0.02	0.97	0.95
Item 10	Sexual activities difficult	0.25	0.02	1.14	1.03
Item 15	Medical costs	0.18	0.01	0.92	0.88
Item 8	Working to earn a living difficult	0.06	0.02	1.21	1.11
Item 9	Recreational activities difficult	−0.31	0.01	0.81	0.78
Item 14	Hospitalization	−0.57	0.02	1.10	1.10

*δ* level of severity (higher values indicate higher severity), *SE* standard error, *MNSQ* mean square fit statistic.

Item separation index of each model: 25.31 for the MLHFQ physical dimension, 21.08 for the MLHFQ emotional dimension, 18.01 for the social dimension proposed by Lambrinou, 14.63 for the social dimension proposed by Garin, 20.96 for the social dimension proposed by Munyombwe, and 27.18 for the MLHFQ total scale

### Comparison of different factor structures

The results of the CFA applied to other factor structures proposed (Appendix 1) are shown in Table 2. The results for the two-factor structure proposed by Heo et al. [7] (hereinafter referred to as Heo’s structure) are satisfactory (RMSEA = 0.08; CFI, TLI > 0.95; range of factor loadings, 0.50 − 0.87). The remaining factor structures considered are three-factor models. Among them, the models proposed by Ho et al. [14] and Moon et al. [4] (Ho’s and Moon’s structures, respectively) obtained unsatisfactory fit, with RMSEA values >0.1, TLIs <0.90, and some factor loadings <0.40. Among the other three-factor models, the best results were obtained with the model proposed by Garin et al. [18] (Garin’s structure). In the models proposed by Lambrinou et al. [21] and Munyombwe et al. [19] (Lambrinou’s and Munyombwe’s structures, respectively), although fit indices were satisfactory, some items showed factor loadings <0.40. Considering the AIC values, the factor structure proposed by the original developers [13] provided the best results among the two-factor models, and Garin’s structure [18] the best results among the three-factor models.

Among the different social factors proposed in the three-factor models, we compared those in Garin’s, Lambrinou’s and Munyombwe’s structures using Rasch analysis (Table 3). The results supported the unidimensionality of Lambrinou’s and Munyombwe’s social dimensions. In the case of the Garin’s social dimension, we found that item 9 had an outfit value slightly below 0.7, and item 15 had both infit and outfit values above 1.3. In all three social dimensions, the item separation index considerably exceeded the minimum required of 2 (18.01, 14.63, and 20.96, respectively). The presence of DIF by sex or age was not detected in any of the social dimensions, and the functioning of the rating scale categories was adequate. Local dependency was found between items 9 and 10 (*r* = 0.51) and items 8 and 10 (*r* = 0.50) in Lambrinou’s social dimension, and between items 8 and 15 (*r* = 0.51) in that proposed by Garin, but not between any items of Munyombwe’s social dimension.

Regarding convergent validity of the social dimensions, SF-12 domain scores were more strongly correlated with Munyombwe’s social dimension than the others (Table 4). Known-groups validity was supported for all social dimensions, patients who attended the emergency department or had any readmissions in the previous 6 months reporting significantly higher MLHFQ social scores (*P* < 0.0001). However, the effect size was much higher for Munyombwe’s social dimension than for the others. Cronbach’s alpha coefficients for the social dimensions proposed by Lambrinou, Garin and Munyombwe were 0.75, 0.71, and 0.76, respectively.

**Table 4:** Correlation between the MLHFQ social subscales and SF-12 components, and known-groups validity of the MLHFQ social subscales

	MLHFQ		
	Social subscale proposed by		
	Lambrinou	Garin	Munyombwe
	ρ coefficient	ρ coefficient	ρ coefficient
SF-12			
Physical Component Summary	−0.24	−0.31	−0.38
Mental Component Summary	−0.31	−0.37	−0.41
	Mean (SD)	Mean (SD)	Mean (SD)
Attended emergency department during the previous 6 months			
Yes (*n* = 239)	7.57 (5.50)	9.58 (6.38)	13.09 (8.54)
No (*n* = 724)	4.98 (5.09)	6.23 (5.92)	7.84 (7.18)
*P* value	<0.0001	<0.0001	<0.0001
Effect size	0.49	0.54	0.67
Readmission during the previous 6 months			
Yes (*n* = 302)	7.29 (5.43)	9.30 (6.40)	13.09 (8.36)
No (*n* = 674)	4.94 (5.10)	6.11 (5.88)	7.42 (6.97)
*P* value	<0.0001	<0.0001	<0.0001
Effect size	0.44	0.51	0.72

ρ: Spearman correlation coefficient, *SD* Standard deviation

Data are expressed as the Spearman correlation coefficient when studying the correlation between the MLHFQ social subscales and SF-12 components, and as the mean (SD) when comparing the MLHFQ social subscales as a function of emergency department attendance, or readmission during the previous 6 months

The scores for the social dimension range from 0 to 15 for that proposed by Lambrinou, from 0 to 20 for that proposed by Garin, and from 0 to 30 for that proposed by Munyombwe, with higher scores indicating worse health status. The scores for the SF-12 dimensions range from 0 to 100, with higher scores indicating better health status

## Discussion

The results of the current prospective study with a large cohort of patients hospitalized for HF at different hospitals support the validity and reliability of the MLHFQ, and most importantly, support the unidimensionality of the MLHFQ total score and the existence of a third factor, a social dimension, with good psychometric properties. To the best of our knowledge, this is the first study that compares different MLHFQ factor structures; this approach is a strength of the research in that it helps us to explore whether the original MLHFQ factor structure is valid, and to assess which of the different social factors proposed is the most appropriate.

Another strength is that we have conducted a complete study of the structural validity, using both confirmatory techniques of CTT, such as CFA, and IRT-based Rasch analysis. Most studies have assessed the structural validity of this questionnaire from the perspective of CTT [4, 7, 16–19, 21, 22], and more specifically, using EFA rather than CFA techniques. Once an instrument has been translated into another language and culturally adapted for the target population, its structure should be confirmed by CFA. We only found two studies in which CFA was conducted [15, 23], one of them using a sample of just 50 patients [23], and we only found one study on the structural validity of the instrument combining both CTT- and IRT-based methods [18].

Regarding two-factor structures, reviewing MLHFQ validation studies, we identified several problems and weaknesses. Specifically, several authors have questioned the factor structure of the questionnaire [2, 5, 7, 11, 14–20]. Our CFA results indicate that the original structure of the questionnaire does have adequate structural validity. Considering the results of Rasch analysis for the physical factor, we found only item 6 to be misfitting. Munyombwe et al. [18], in the only study in which an IRT model is applied to the questionnaire, did not find this item to be problematic in the Rasch analysis, but unlike us, they detected DIF by sex in item 3. None of the other studies that proposed different factor structures [4, 7, 15, 16, 18, 21] drop item 6 from the physical factor. Further, taking into account the satisfactory results obtained from the rest of the Rasch analysis and the satisfactory CFA results, we do not consider that the identification of this item as misfitting is sufficient reason to conclude that this item should be excluded from the physical dimension. Regarding the emotional MLHFQ dimension, the fit indices from Rasch method support unidimensionality and provide strong evidence of construct validity. Munyombwe et al. [18] also found satisfactory results in the Rasch analysis applied to this dimension.

Concerning different factor structures that have been proposed [4, 7, 15, 16, 18, 21], in general, there is consensus about the emotional factor, all but one study agreeing on the constituent items [21]. The largest discrepancies are related to the items that make up the physical factor, and the fact that three-factor structures have emerged in some studies, the new factor corresponding to a social dimension [4, 15, 16, 18, 21]. In relation to the two-factor structures considered, Heo et al. [7] proposed a physical factor which includes the same items as the original developers and adds two more items, item 1 and item 9, maintaining the same emotional factor. Other authors have also proposed that item 1 be included in the physical factor [4, 15, 16, 18]; however, when comparing our CFA results for Heo’s model with those for the original model [7], we obtain slightly better results for the latter. Hence, we rule out Heo’s model as an alternative to the original.

In relation to the physical factor suggested by other authors (Appendix 1), Ho and Moon both proposed a factor with somewhat larger discrepancies with the original. Further, we found the worst CFA results for these two proposals. Among the other structures, the composition of the physical factor differs only in one or two items. However, as noted previously [15], the modification of an instrument is not easy. Besides, in the case of this questionnaire, the new structures that have been proposed are generally obtained from EFA and not CFA [15], and on the other hand, the widespread use of the questionnaire means that changes would be difficult to implement and would also hinder comparability with existing data. Consequently, and considering that the results from both CTT and IRT for this factor were satisfactory, we see no need to establish a different composition for the physical factor.

With respect to a potential third factor, representing a social dimension, adding a third factor would not be as complicated as changing the composition of existing factors, since it would not involve any change to what was established by the original developers [13] or affect comparability with other studies. However, it is important to reach a consensus on which of the different social factors proposed is the most appropriate and has the best psychometric properties [4, 15, 16, 18, 21]. Although several authors have proposed such a third factor, none of them have studied the properties of the factor from the perspective of IRT, or using confirmatory techniques. In our analysis, the Ho and Moon social factors were considered inadequate, having fit indices below the minimum required, and obtained the highest AIC values. Furthermore, they included items of the physical factor proposed by the original developers in their social factor, implying a complete change of structure. The remaining proposals for a social factor only disagree on a few items. All of them considered items 8, 9 and 10; Garin also included item 15; and Munyombwe, besides item 15, includes items 14 and 16. Regarding the results of the CFA, the lowest AIC value was obtained for Garin’s factor. However, to compare the three social factors, it is also necessary to consider the IRT results, because in the CFA we are analyzing the complete structure of the questionnaire and not just the social factor. Rasch analysis results are satisfactory for all three structures, although Munyombwe’s model is the only one that met all the requirements to be considered an acceptable model. Further, regarding convergent validity, known-groups validity, and reliability, the best results were found for Munyombwe’s social dimension, with the highest correlation coefficients with the SF-12 components, the highest ES in known-groups validity, and the highest Cronbach’s alpha coefficient.

Lastly, the MLHFQ is commonly used to generate a total score, which assumes that the total scale is unidimensional. However, we found only two previous studies [15, 18] that had explored the existence of a single factor, and they differ in their conclusions. The first one [15] applied CFA within a bifactor model and the results confirmed the unidimensionality of the total score. The other study [18] applied Rasch analysis to study the dimensionality of the total factor, and authors concluded that there were some misfitting items, namely, items 7, 8, 10, 14 and 15. They also found DIF by age in items 1 and 8, and by sex in item 3. Regarding misfitting items, Heo et al. [7] concluded that items 8, 10, 14, 15 and 16 were problematic. Another study [44] also stated that items 8, 10 and 15 were problematic, since they were not applicable to all patients. In our case, we only found two items to be markedly misfitting, items 8 and 10, with infit and outfit values well above the threshold. In item 1, we also found some degree of misfit with an outfit of 1.82, and in item 10, DIF was detected by sex and age, men and younger patients finding this item more difficult than women and older patients. Therefore, we confirm the existence of some problematic items in the composition of the total score, but unlike some previous authors [18], we did not detect problems in the functioning of the rating scale categories. As Munyombwe et al. [18] stated, the fact that there are misfitting items does not necessarily imply the need to remove them from the questionnaire, above all when these items would be included in the social factor. Considering a third factor, most of the 21 items would be included in a factor, and hence considering factor scores for the three-factor structure could be an alternative to the total score.

This study has some limitations that should be taken into account. The sample is composed of patients in Spain and we used the Spanish version of the questionnaire, and hence the results may not be generalizable to other populations or other language versions. Moreover, besides having to be valid and reliable, an instrument must also be responsive to changes to be useful. To the best of our knowledge, although there are some studies on the responsiveness of physical, emotional and total scores, the responsiveness of the social factor proposed has not yet been explored.

## Conclusions

In conclusion, this comprehensive validation process, which used a large patient sample and combined classical and contemporary methods, supports the validity of MLHFQ physical and emotional subscales in patients with HF, showing good properties from both CTT- and IRT-based perspectives. In addition, the results confirmed the existence of a third factor, and we recommend the use of Munyombwe’s social factor, since it has good psychometric properties, the best among the social factors proposed. On the other hand, we found some problematic items within the total score, implying that it should be used with caution. Moreover, given the validity of the social factor, 19 of the 21 items would be included in a factor, and consequently, factor scores for the three-factor structure could be an alternative to the total factor. In conclusion, this study provides strong evidence that the MLHFQ is useful for measuring HRQoL in patients with HF, and it can be used both in clinical practice and research.

### Ethics, consent and permissions

The study was approved by each corresponding institutional review board. All patients were given a letter informing them about the study and asking for their voluntary participation.

## Appendix

**Table 5:** Summary of MLHFQ different factor structures proposed

First author	Physical		Emotional		Social	
	No. of items	Items	No. of items	Items	No. of items	Items
Rector, 1992 [10]	8	Q2, Q3, Q4, Q5, Q6, Q7, Q12, Q13	5	Q17, Q18, Q19, Q20, Q21		
Heo, 2005 [7]	10	Q1, Q2, Q3, Q4, Q5, Q6, Q7, Q9, Q12, Q13	5	Q17, Q18, Q19, Q20, Q21		
Ho, 2007 [16]	8	Q1, Q2, Q3, Q6, Q11, Q12, Q13, Q16	5	Q17, Q18, Q19, Q20, Q21	6	Q4, Q5, Q7, Q8, Q9, Q10
Moon, 2012 [4]	12	Q1, Q2, Q3, Q4, Q5, Q6, Q9, Q10, Q11, Q12, Q13, Q16	5	Q17, Q18, Q19, Q20, Q21	4	Q7, Q8, Q14, Q15
Lambrinou, 2013 [21]	11	Q2, Q3, Q4, Q5, Q6, Q7, Q9, Q11, Q12, Q13, Q14	6	Q1, Q16, Q18, Q19, Q20, Q21	3	Q8, Q9, Q10
Garin, 2013 [15]	8	Q1, Q2, Q3, Q4, Q5, Q6, Q12, Q13	5	Q17, Q18, Q19, Q20, Q21	4	Q8, Q9, Q10, Q15
Munyombwe, 2014 [18]	9	Q1, Q2, Q3, Q4, Q5, Q6, Q7, Q12, Q13	5	Q17, Q18, Q19, Q20, Q21	4	Q8, Q9, Q10, Q14, Q15, Q16

## Abbreviations

AIC: Akaike Information Criterion
CFA: Confirmatory factor analysis
CFI: Comparative Fit Index
CTT: Classical test theory
DIF: Differential item functioning
EFA: Exploratory factor analysis
HF: Heart failure
HRQoL: Health-related quality of life
INFIT: Mean square information-weighted statistic
IRT: Item response theory
MCS: Mental Component Summary
MLHFQ: Minnesota Living with Heart Failure Questionnaire
OUTFIT: Outlier-sensitive statistic
PCA: Principal component analysis
PCS: Physical Component Summary
RMSEA: Root mean square error of approximation
SD: Standard deviation
SF-12: 12-item Short Form Health Survey
TLI: Tucker-Lewis Index
